# Cerebral Hyperæmia

**Published:** 1883-10

**Authors:** J. M. G. Carter

**Affiliations:** Waukegan, Illinois


### ARTICLE III.

**CEREBRAL HYPERÆMIA.** By J. M. G. CARTER, A.M., M.D. Waukegan, Illinois.

The increased flow of blood to the brain, commonly called cerebral hyperæmia, or congestion of the brain, is a condition of very frequent occurrence, and the cause of much physical and mental suffering. It exhibits numerous forms, and depends upon a variety of causes.

Many varieties of cerebral congestion have been described, and elaborate classifications made, but in practice they are usually grouped under two heads—acute and chronic. Congestion may occur in various degrees of intensity, from a slight increase above the normal cerebral circulation to such an influx of blood to the brain as will entirely destroy its functions. In whatever degree of severity the attack is made, if the onset is sudden and of short duration it is acute. If, on the other hand, the morbid process is slow in its progress and the disease of long continuance, the case is chronic. It is evident that the chronic variety may result from the acute, and the acute may supervene upon the chronic. In the chronic form the impairment of cell function is more gradual, stupor is more characteristic, and there is a greater amount of extravasation from minute vessels. In the acute form there is greater tendency to rupture of vessels and sudden loss of consciousness. The following cases from private practice illustrate these forms, and show the usual course and symptoms of the disease :

J. C., æt. 26, was a close student and a hard worker, by profession a teacher. About the middle of May, 1872, he first felt a pain in the right superior frontal region, which interfered materially with mental application, or close attention to any subject or work. Every day toward the close of his work there was a heavy, uneasy sensation of fullness in the head, not unfrequently attended with pain. Occasionally he was annoyed by muscular twitchings of the eyelids, specks floating before the eyes, ringing in the ears, vertigo, and the like. These symptoms continued until the close of his school in the latter part of June. The sensations of tightness, like a band surrounding the head, dread of some impending evil, delusions and hallucinations, so often present in this variety, were not present, or, if so, in so slight degree as not to be noticed.

By the close of his school the symptoms were more marked. The pain was sharper than at first, and occurred more frequently during the day. His sleep was generally good after the first hour or two, and sometimes there seemed to be no insomnia even during the first hour after retiring.

When the pain was most severe the blood-vessels about the neck and head were distended and throbbing, and this always occurred during or after close mental application.

A short rest from his studies greatly relieved his troubles. Soon, however, he felt that it was necessary for him to resume his labors. No sooner had he commenced his work than there was a return of the disturbance of the cerebral circulation—pain, dizziness, full carotids, and " nervousness." These symptoms increased, until the first of October he was almost prostrated, notwithstanding his excellent appetite and care about physical exercise. He was compelled to desist from both mental and physical labor, or exertion of any kind, not being able to do more than take very simple recreation, on account of the disturbance of the cerebral circulation which it invariably caused. He consulted a physician. For four months his condition remained unimproved. He was taking phosphorus during the entire time, and once or twice applied a Spanish-fly blister to the nape of the neck. He was unable to read more than five or ten minutes at a time without bringing on severe pain in the head, and physical exertion, as sawing wood, was just as sure to produce pain.

Finally, in February, 1873, the patient thought it advisable to secure the services of another physician. The trouble was diagnosed chronic congestion of the brain, with an atonic condition of the cerebral vessels. Rest, nourishing food, moderate out-door exercise were enjoined; and the pyrophosphate of iron with calisaya bark was prescribed as a tonic.

In four weeks the patient's condition was so much improved that, with the consent of his physician, he returned to teaching. By continued use of the tonic he was able to do the work of the schoolroom; but it was some four years before he was able to perform the amount of intellectual labor that he was accustomed to do before the commencement of his trouble in May, 1872.

T. S. W., æt. seventy-two, a Scotchman, of robust constitution; by occupation a farmer; temperate and of regular habits. In February, 1883, was thrown from a wagon, but not seriously injured. After that accident his family noticed that he was more irritable, sometimes peevish and fretful. His decisions were not so pronounced, and perhaps would be reversed several times before he settled on a line of action. He did not feel as well as before, and gradually discovered that he was not able to work as formerly. He had a terrible dread of impending financial disaster, notwithstanding he had a good farm and was surrounded by plenty.

About the 1st of July his symptoms were perceptibly worse, and he was disturbed by dizzy spells. July 8, while hoeing in the field he was suddenly seized with dizziness and fell forward to the ground. With some difficulty he got up and went to the house. Next day there was a recurrence of the trouble, and he had greater difficulty in getting up. Having been sent for to see another member of the family, the physician was consulted in regard to Mr. W.

At that time, July 10, his pulse was slow—56—and full. There was a constant pain in the right superior frontal region, which occasionally extended across to the left side of the head. The bowels, which had been loose, were costive, and the tongue was coated. The carotids were distended, and the eyes somewhat congested, especially the retina.

Here was evidently a case of chronic congestion of the brain, with an impending acute attack. Rest, quiet, arterial sedatives, bromides and cold to the head were prescribed, and an engagement left for next day.

July 11. Found that he had been out; had fallen and was unable to get up without help. The pulse 54; pupils contracted; intellect somewhat dull; sensibilities obtunded; bowels moving freely from action of purgatives. Second visit: Pulse 60; other symptoms the same, but patient more restless. Chloral and ergot added to treatment.

July 12, first visit: Pulse 60; temperature normal; pupils contracted; patient restless and somewhat delirious; bowels constipated; appetite gone; motion on right side impaired. Second visit: Symptoms aggravated; pulse 80; temperature 101°; right side paralyzed, bowels constipated; pupils not changed.

July 13. Symptoms aggravated; patient unconscious; bowels had moved several times; breathing heavy; patient very restless; urine high-colored and scanty.

July 14. Patient in about the same condition as yesterday. Second visit: Pulse variable; urine passed involuntarily, high-colored and scanty.

July 15. Aroused with difficulty; great difficulty in swallowing; food ordered by enemata; pulse variable, ranging from 60 to 85; temperature about 101°; pupils variable, but generally small, yet affected by the action of light. Second visit: More easily aroused; coma deepening, except when wakened.

July 16. Aroused, and called the family, recognizing each member: pulse more regular at 80; food taken through stomach; between the lucid intervals the breathing was very heavy, almost stertorous ; the heavy coating which had covered the tongue had disappeared, leaving the surface moist and clean. Second visit: No particular change ; patient apparently better ; urine more abundant, but high-colored.

July 17. Patient was restless through last night ; swallowing more difficult ; not so many lucid intervals ; other symptoms about the same. Second visit : Coma deepening.

July 18. Patient restless and unconscious ; took very little food ; pulse variable and weak ; temperature variable, not reaching more than 102° ; tongue drops into back of mouth as if paralyzed; patient turned on one side to facilitate breathing ; coma becoming deeper and respirations more shallow.

Second visit—Deep coma ; pulse variable and weak, ranging between 85 and 100 ; temperature unchanged ; pupils contracted, yielding less to the influence of light ; has taken no nourishment and cannot swallow ; sinking rapidly ; July 19, died at 2:15 A. M.

The face was not affected by paralysis, and when the tongue was protruded it did not deviate to either side. The muscles of the tongue seemed to be slightly involved as a whole, as it was with difficulty that the patient protruded it, and his speech during his lucid moments was difficult and his syllables were slurred, and a sort of thickness was noticeable in his pronunciation. The paralysis on the right side was total as regarded motion, and partial as regarded sensation. This is what Dr. Hammond calls the *apoplectic form.* It is so denominated in Reynold's System of Medicine, and by other authors. This form is most common in advanced life, and is generally fatal.

P. D., æt. 25, a robust farmer ; had been working in the sun during the forenoon, and in the afternoon rode to town. The effect of the heat made him dizzy, and hoping to feel better, he took a glass of beer. Soon he went into the sun again, was seized with a feeling of dizziness and fell unconscious. Medical aid was called immediately.

The pulse was 60, temperature normal, head hot, and patient unconscious. At first there were suspicions that it was a case of intoxication, but careful examination of the symptoms and history showed it to be a case of acute congestion of the brain produced by the combined effect of the heat and beer. The free use of arterial sedatives and cold applied to the head restored consciousness, and in two days the patient was quite well.

It is important to know that many school children suffer from cerebral hyperæmia. It is also true that many children and parents complain of the over-taxing work assigned at school, when in fact the trouble is due to dissipation, late hours, novel reading, and the like, and is not at all due to excess of school work. Nevertheless, there are many cases of this malady among the children of our public schools.

A. G. was a healthy, rosy-cheeked girl of twelve years. She was in a class of boys and girls from fourteen to sixteen years of age, and being very ambitious, she studied very hard that she might excel. She soon became the subject of headaches, which increased in severity and frequency of recurrence. They were accompanied by flushing of the face, full carotids, redness of the eyes, and a sensation of fullness or tightness in the head. They did not trouble her on rest or holidays, but finally invariably followed close mental application. At times she was troubled with wakefulness; her appetite became capricious, and it became necessary to take her away from school. Rest, nourishing food, and moderate exercise restored her to health.

Any close mental application may bring on cerebral congestion. Drinking spirituous liquors, the use of certain drugs, especially opium, and the excessive use of tea and coffee, may lead to the same result. Extremes of heat and cold, malaria, renal, cardiac and other diseases predispose to this malady.

To understand the action of these ætiological agents, and to grasp the pathology of this disease, it is necessary to examine some points in the physiology of cerebral circulation under normal circumstances. About one-fifth of all the blood in the body passes through the brain at each cycle of the circulation. Hence when the heart's action is increased in rapidity the brain receives in a given time an increased quantity of blood.

Now, considering the small size of the brain and the delicate structure of its tissues, it is evident that an increase in the heart's action must throw a relatively enormous amount of blood into the encephalon. The blood passes to the brain with greater velocity than it does to the rest of the body; for, in accordance with the principles of mechanics, *fluids driven with the same force of compulsion* will flow more rapidly through small tubes (above capillary size) than through tubes of large caliber. Dr. Fleury has shown that in conformity to this law, with the aid of the power of vital contraction and the natural lubrication of the vessels, the blood in the carotids flows with greater velocity than it does in the aörta. (*Du Dynamisme Comparé des Hémisphères Cerebraux chez L'Homme.*) He also shows that there is a greater quantity of blood going to the left hemisphere than to the right. This depends partly upon the law just given and partly upon the anatomy of the parts. As the brachio-cephalic, or in-nomniate artery rises from the arch of the aörta, it inclines toward the right. Hence, when the current of blood from the heart arrives at this point, that portion which passes into the in-nominate artery must turn somewhat backward; and the result is that a smaller current enters this vessel than would if it arose from the arch inclining to the left of the linear axis of the body. Again, the current evidently strikes upon the left or internal wall of the vessel, and must be deflected toward the right, and the effect of this is to allow a smaller quantity of blood to pass into the common carotid than would pass into that vessel if the channel were straight, the whole amount being less than half what enters the brachio-cephalic.

The left common carotid is half at large as the innominate but at its origin from the arch of the aörta it inclines toward the left, so that it receives more than half as much blood as passes into the innominate artery. In accordance with the mechanical law before stated, the velocity of the blood current in the left carotid is greater than that in the innominate, and likewise it is greater than the velocity of the current in the right carotid. Thus it is readily seen that there is a greater quantity of blood passing to the left hemisphere of the brain than to the right. In this fact we have not only the reason for the superiority of the left hemisphere over the right in functional activity, but an explanation also of the greater frequency of hæmorrhage into the left side of the brain. Reference to these anatomical and physiological facts leads to a rational explanation of the paralytic phenomena occurring on the right side, accompanying cerebral congestion so frequently. Any cause which increases the flow of blood to the brain necessarily produces greater disturbance on the left side of that organ; for, as the actual amount of blood received is greater than that received by the right hemisphere, the structures of the left hemisphere must be subject to greater blood pressure in congestion than the opposite side, and of course the tendency to rupture is greater. There is likewise greater danger of aneurism, or softening from pressure. Remembering, then, the fact of the crossing of the motor nerves in the medulla oblongata, the oft repeated question, "Why does paralysis most frequently occur on the right side?" is easily answered.

After the blood reaches the brain it passes into extremely small and delicate capillaries. Here any slight influences may interfere with the circulation in the encephalon.

An increase in the amount of blood in the brain may depend upon one or more of at least five causes—gravitation, obstruction, mental activity, physical activity, and vaso-motor paralysis.

If the hyperæmia depend upon either of the first two, it will be arterial; and if it depend upon the last, it may be either venous or arterial, or both.

Congestions produced by the stooping position, as in certain occupations, are due to gravitation. Tumors in the course of veins returning blood from the head, and other similar influences, act as obstructions to the venous flow of the blood, and produce venous congestion. Any activity, whether mental or physical, increases the flow of arterial blood to the brain, and when excessive, produces congestion.

The vaso-motor nervous system is that system of nerves and ganglia whose chief function, so far as known, is to control the caliber of the blood-vessels. Stimulation of these nerves produces contraction of the vessels, and paralysis produces dilatation. Drugs, like alcohol or opium, paralyze the vaso-motor nerves, and as a consequence produce dilatation of the vessels.

Whichever of the five causes named is the means of producing a congestion, the effects are the same. The first result, if the cause acts to excess, is distension of the vascular walls. The distension produces pain by pressure. A continuation of the cause tends to induce an atonic condition of the vessels by paralyzing the vaso-motor nerves, or destroying the normal tonicity of the vascular walls. This is the condition usually present in cases of chronic cerebral hyperæmia.

When the blood is most abundant in the brain, the mental processes are most active, and this causes wakefulness and increased power of imagination. But when the brain is most active, waste is also most active, and there is need of more rapid repair. If waste exceed repair, there will result both atrophy of the part affected, and functional inactivity. Softening of the brain tissue is a frequent result. According to the mechanical law given above, if the vessels become dilated, the blood current is retarded in its flow ; and Traube has found in the retardation of the blood current a source of sclerosis of the arteries. The white corpuscles, he says, remaining adherent to the lining membrane of the vessels, become changed into connective tissue, and finally undergo fatty degeneration or calcification. (Karl Hertzka, *Journal of Nervous and Mental Disease*, July, 1875.) The atheromatous procees which follows this retardation may extend into the smallest cerebral arteries, and result in miliary aneurisms. In this way may be caused the pareses and paralyses which frequently result in cases of chronic cerebral congestion.

Furthermore, by continued distension of the capillaries, the perivascular spaces become filled out, and this prevents the circulation of the finest lymphatic vessels. This interstitial fluid expands other spaces, especially connective tissue cells. These cells enlarge and undergo a division of the nuclei, and the proliferation results in sclerosis of the brain. (Ibid.)

I have no doubt that this is the correct explanation of many of the cases of "nervousness" and "shaking palsy" in patients who have overworked, or who have been addicted to the use of alcoholic drinks or other intoxicating drugs.

The malarial poison increases the susceptibility of the various tissues of the body. This renders the organs more easily affected by an increased flow of blood, and more easily exhausted by overwork. In view of the fact that visceral congestions generally attend malaria, it may be concluded that the vaso-motor system is affected, and may be paralyzed, by this poison. There is no reason why the persistent action of this agent may not produce congestion of the brain as well as of the liver, or any other organ; especially if the brain be subjected to as much abuse as the digestive apparatus; nor does it matter whether the poison is a blood destroyer or vaso-motor depressor.

The prolonged action of the malarial poison, like the prolonged action of the other causes of congestion, can only result in chronic cerebral hyperæmia. Such is the case with old whisky-drinkers and opium-eaters.

The pathological condition resulting from the use of particular drugs must be due to vaso-motor paralysis or to an increased action of the heart. These influences, continued for months or years, can only terminate in chronic congestion of the brain. The condition itself is first distension, then pressure upon the brain tissue, and at last sclerosis; or the vessels are dila'ed, tonicity is lost, retardation of the blood current follows, the atheromatous process sets in, and aneurism or rupture may result, accompanied by pariesis or paralysis, and, perhaps, ending in softening of the brain and death.

Köser has shown that the blood of overworked or hunted-down animals is poisonous, rendering the flesh of such animals destructive to life. The same must be true, to a certain extent, of the blood of overworked men. Such blood cannot be proper food for the tissues, and particularly such tissues as the brain. This may be another influence to hasten the atheromatous process in the cerebral vessels. A great deal might be said concerning the treatment of this malady, but a few words must suffice here, as the text-books are sufficiently explicit on that point.

For an acute cerebral congestion the indication is to cool the head and diminish the quantity of blood in the distended vessels. Cold should be applied to the head in the form of ice or very cold water. Ergot and bromides diminish the caliber of the blood vessels, and hence reduce the quantity of blood in the brain. Arterial sedatives control the action of the heart, and prevent the flow of so much blood into the cranial cavity as would otherwise reach it.

In the chronic form, where the vessels have lost their tone, iron, strychnia and phosphorus will meet the indication. If iron cannot be borne, arsenic may be substituted for it. Where malaria has had anything to do with the case, iron and quinine, or arsenic and quinine, are indicated. Where neither iron nor arsenic can be borne it may be advisable to use zinc. If an anodyne is needed, chloral hydrate should be used. In the chronic form, moderate exercise, nourishing food, and bathings are necessary.

